# Clinicoepidemiological Observations of Enteric Fever in Infants: Experiences From a Tertiary Care Pediatric Hospital in North India

**DOI:** 10.1093/infdis/jiab430

**Published:** 2021-11-23

**Authors:** Karnika Saigal, Deepika Gupta, Diganta Saikia

**Affiliations:** 1 Department of Microbiology and Infectious Diseases, Chacha Nehru Bal Chikitsalaya, New Delhi, India; 3 Department of Pediatrics, Chacha Nehru Bal Chikitsalaya, New Delhi, India

**Keywords:** *Salmonella* Typhi, *Salmonella* Paratyphi A, enteric fever, case-fatality rate, surveillance

## Abstract

**Background:**

There is a lack of evident data to explain the true scenario of age-specific enteric fever in India. The current study aimed to evaluate the burden and disease pattern of enteric fever among infants in a tertiary care pediatric hospital.

**Methods:**

A prospective laboratory-based surveillance was conducted from April 2018 to January 2020 at a children’s hospital in North India, under the Surveillance for Enteric Fever in India study. The study included children <1 year of age in whom *Salmonella* serovar Typhi/*Salmonella* serovar Paratyphi grew in cultures from blood or sterile body fluid. The key outcome measures included disease spectrum and clinical presentation.

**Results:**

Of the 10 737 blood cultures from infants, 26 were positive for *S.* Typhi or *S.* Paratyphi. The majority of cases occurred in infants aged 6–12 months, with the youngest being 1 month old. Fever with abdominal pain and diarrhea were the common symptoms, with 46% of infants requiring inpatient care. All of the isolates were susceptible to ceftriaxone. Third-generation cephalosporins were used as the first-line therapy for hospitalized infants. The average duration of fever was 8.6 days. The overall case-fatality rate among infants with enteric fever was 7.4%.

**Conclusions:**

Enteric fever is a major contributor to disease and death among children. Robust surveillance studies are required to understand the true disease burden.

Enteric fever is a life-threatening systemic infection caused by *Salmonella enterica* serotype Typhi and *S. enterica* serotype Paratyphi (serovars A, B, and C). The incidence, disease burden, and case-fatality rates of enteric fever show substantial variation with geographic distribution, seasonality, age at presentation, and resistance profile of the organism isolated. Enteric fever remains a persistent public health problem in low- and middle-income countries [1, 2]. This was made evident by the Global Burden of Disease study (2017), which estimated 14.3 million cases of enteric fever and 135.9 thousand deaths occurred globally in 2017 [3], with a majority attributed to low- and middle-income countries in South Asia, Southeast Asia, or sub-Saharan Africa. The case-fatality rate is reported higher in children <5 years of age than in school-aged children and adolescents, particularly in sub-Saharan Africa and North Africa/Middle East regions [4–7]. Moreover, the growing burden of drug-resistant strains are cause of global concern. This disproportional burden stresses the need for controlling the disease in these regions.

In India, very few epidemiological studies have documented the age-specific incidence of typhoid fever, with the estimated incidence of typhoid fever 377 per 100 000 person-years. The highest incidence of enteric fever is reported among children aged 2–4 years [8, 9]. The World Health Organization (WHO) recommends the introduction of typhoid conjugate vaccine (TCV) into routine immunization programs in high-burden countries, including India [10].Without much data, the country is left with large gaps in its understanding of vaccine target groups. To help fill these gaps, the current study aimed to evaluate the burden, disease pattern, and prevailing antimicrobial susceptibility pattern of enteric fever among infants in a tertiary care pediatric hospital in India.

## METHODS

### Study Design, Area, and Time Period

This prospective, laboratory-based surveillance study was conducted from April 2018 to January 2020 at Chacha Nehru Bal Chikitsalaya, a tertiary care children’s hospital in New Delhi, India, as part of the third tier of the Surveillance for Enteric Fever in India network. Chacha Nehru Bal Chikitsalaya offers comprehensive healthcare for the pediatric population in Delhi and adjoining states, along with tertiary care and referral services for patients from across North India. The hospital primarily serves low– to middle–socioeconomic status populations.

### Study Population

Included in this study were children <1 year of age who were treated in the hospital’s outpatient or inpatient services and who had blood or sterile body fluid cultures in which *S.* Typhi or *S.* Paratyphi grew.

### Data Collection

Eligible patients were enrolled after written informed consent was obtained from the parent or legal guardian. On patient enrollment, demographic details, clinical information and history of current disease, vaccination status, and culture findings were recorded prospectively in the predesigned case report form by research staff. In addition, risk factors such as mode and place of delivery, mode of feeding, and prior hospitalization were also recorded. The demographic features of enrolled case patients were obtained from caregivers, and clinical information—history (including treatment received before admission), physical findings, diagnosis, and treatment prescribed—was recorded from the admission case sheets and verified with the pediatrician.

### Sample Collection

Blood and/or other sterile body specimens were collected in preweighed Peds Plus BACTEC bottles (FX200; Becton Dickinson) by aseptic means, per standard protocol. Bacterial identification and antimicrobial susceptibility testing were performed using matrix-assisted laser desorption ionization time-of-flight mass spectrometry and a Vitek2C system. Antimicrobial susceptibility testing, using antibiotic discs of ampicillin (10 μg), chloramphenicol (30 μg), azithromycin (15 μg), and pefloxacin (5 μg), was performed via the Kirby-Bauer disk diffusion technique, and results were interpreted as sensitive, intermediate, or resistant, according to Clinical and Laboratory Standard Institute (M100-S25) 2015 criteria [11]. Standard operational procedures guidelines were followed during sample collection and processing.

American Type Culture Collection (ATCC) reference strains of *Escherichia coli* (ATCC-25922) and *Staphylococcus aureus* (ATCC-25923) were used to check the quality control culture medium and antimicrobial disks. The isolates were serotyped using *Salmonella* antiserum, per the manufacturer’s instructions (Denka Seiken).

### Data Processing and Analysis

Data were collected, coded, and entered into Web-based software on a daily basis. The exported data from the Web were analyzed using Statistical Package for the Social Sciences software (SPSS; version 16).

### Ethical Considerations

The study protocol was approved by the ethical committees of the Indian Council of Medical Research, Christian Medical College Vellore, and the institutional ethical committee, Maulana Azad Medical College (F.1/IEC/MAMC [60/01/2018/No. 224]). Written informed consent was obtained from a parent or guardian for all children in the study .

## RESULTS

During the study period, a total of 33 086 blood cultures from children <12 years of age were submitted to the laboratory for bacteriological investigation, from which 624 isolates of *Salmonella* species and 10 nontyphoidal *Salmonella* species were isolated (Table 1). Of the 10 737 blood cultures from infants, 26 were positive for *Salmonella* species. Twenty-five infants (96%) were infected with *S. enterica* Typhi, and 1 with *S. enterica* Paratyphi A.

**Table 1.** Age Distribution of Patients With Laboratory-Confirmed Enteric Fever

Patient Age Group, y	Blood Cultures, No.	Enteric Fever Cases, No.
0–12	33 086	624^a^
<1	10 737	26^b^
>1 to 2	6376	111
>2 to 5	8330	205
>5	7643	282

^a^Of 624 *Salmonella* isolates, 622 were from blood and 2 from cerebrospinal fluid cultures.

^b^Of 26 positive isolates, 2 were from cerebrospinal fluid cultures.

Of these 26 infants, 54% (n = 14) were treated as outpatients, and 46% (n = 12) required inpatient care. *S*. Typhi was isolated from the blood in a majority of the patients (96%), except in one presenting with features of meningitis, in whom it was isolated from cerebrospinal fluid as well. *S.* Paratyphi A was isolated from blood culture in one patient, a 9-month-old infant.

The burden of enteric fever among infants aged 1–3, >3 to 6, >6 to 9, and >9 to 12 months was 19%, 8%, 38%, and 35%, respectively (Table 2). The majority of typhoidal infections occurred in infants aged >6 to 12 months (n = 19, 73%), with the youngest being 1 month old. The clinical profile of infants presenting with typhoid fever, clinical outcome, and antimicrobial resistance pattern was further evaluated (Table 3). The mean weights of children aged 1–3, >3 to 6, >6 to 9, and >9 to 12 months were 3.5, 5.2, 7.3, and 8.2 kg, respectively. Of the 25 infants for whom the mode of delivery was available, 84% had vaginal and 16% had cesarean deliveries. The gestational age was available for 24 infants, all of whom were delivered at full term. Of the 23 infants for whom feeding practices data was available, 61% (n = 14) were bottle fed, and 39% (n = 9) were exclusively breastfed.

**Table 2.** Age Distribution of 26 Infants With Laboratory-Confirmed Enteric Fever

Patient Age, mo	Infants, No. (%)
1–3	5 (19)
>3 to 6	2 (8)
>6 to 9	10 (38)
>9 to 12	9 (35)
**Total**	**26 (100)**

**Table 3.** Risk Factors, Clinical Presentation, and Outcome Among Infants With Laboratory-Confirmed Enteric Fever

Characteristic or Outcome	Infants, No. (%) (n = 26)^a^
Age, mo	
<5	7 (27)
>5 to <12	19 (73)
Sex	
Male	13 (50)
Female	13 (50)
Weight, mean, kg	
At time of disease	6.88
Birth weight	2.95
Hospitalization status	
Inpatient	12 (46)
Outpatient	14 (54)
Mode of delivery (n = 25)	
Vaginal	21 (84)
Cesarean	4 (16)
Gestational age: full term (n = 24)	24 (100)
Feeding method (n = 23)	
Breastfed	9 (39)
Bottle fed	14 (61)
Mother with history of enteric fever	2 (8)
Symptoms at presentation	
Fever (n = 26)	25 (96)
Diarrhea (n = 23)	10 (43)
Vomiting (n = 23)	10 (43)
Cough (n = 23)	9 (39)
Abdominal pain (n = 23)	4 (17)
Breathlessness (n = 23)	1 (4.3)
Seizure (n = 23)	1 (4.3)
Other^b^ (n = 23)	5
Final outcome among inpatients (n = 12)	
Discharged	9 (75)
Left against medical advice	1 (8.3)
Death	2 (16.7)
Overall case-fatality rate	2 (7.6)
Length of stay, mean, d	10
Fever defervescence time, mean, d	8.6

^a^Data represent no. (%) of infants unless otherwise specified. Unless otherwise indicated in the row heading or subheading, total n = 26.

^b^Other symptoms at admission included decreased oral intake and lethargy in 3 patients and abdominal distension, nasal discharge, ear itching, and body ache in 1 patient each.

Complete clinical records and outcomes were available for 23 patients (Tables 3 and 4). The most common presentation for both inpatients and outpatients was fever with gastroenteritis. Inpatients also showed features of dull or decreased activity, decreased oral intake, and abdominal distension (Table 4). The mean hemoglobin level for all 26 infants was 9.6 g/dL. According to WHO guidelines to children aged 5 months to 5 years, mild (hemoglobin, 10–10.9 g/dL), moderate (7–9.9 g/dL), or severe anemia (<7 g/dL) was observed in 6, 14, and 1 patient, respectively. Abnormal liver enzyme levels were noted in 16 patients.

**Table 4.** Clinical Presentation Among Infants With Enteric Fever Requiring Inpatient Versus Outpatient Care

Symptom at Presentation	Infants, No. (%)	
	Inpatients (n = 12)	Outpatients (n = 11)^a^
Fever	11 (92)	11 (100)
Diarrhea	6 (50)	4 (36)
Vomiting	6 (50)	4 (36)
Cough	6 (50)	3 (27)
Abdominal pain	2 (17)	2 (18)
Breathlessness	1 (8)	0
Seizure	0	1 (9)
Other features		
Decreased oral intake	2 (17)	0
Dull, decreased activity	1 (8)	0
Abdominal distension	1 (8)	0
Nasal discharge	0	1 (9)

^a^Clinical data were not available for 3 outpatients.

The mean duration of stay among the inpatients was 10 days, and 50% had a stay of >7 days. A majority of inpatients (75%) recovered and were discharged. The overall case-fatality rate among infants with enteric fever was 7.6% (2 of 26). The first fatal case occurred in a female infant, born at full term by cesarean delivery and presenting at age 1 month with fever, decreased oral intake, and decreased activity for the past 24 hours. The infant died within 48 hours of hospital admission. The second fatal case was in a 7-month-old boy admitted with high-grade fever, difficulty breathing, and decreased activity for the past 24 hours. A final diagnosis of sepsis with pneumonia was made, and the infant died within 24 hours of hospital admission.

A follow-up visit on day 28 day after enrollment was done to review the outcome in inpatient enteric fever cases. Of the 9 discharged patients, 8 were found to be healthy at this visit, and 1 was lost to follow-up.

**Figure 1.**
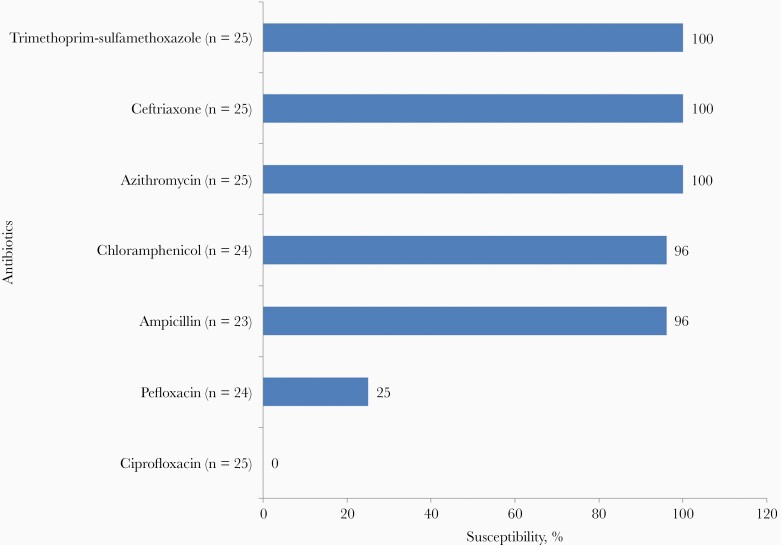
Percentage susceptibility of *Salmonella* Serovar Typhi for various antimicrobials.

All the *S*. Typhi isolates were susceptible to ceftriaxone, azithromycin, and trimethoprim-sulfamethoxazole (Figure 1). However, none of the isolates were found to be susceptible to ciprofloxacin. The single *S.* Paratyphi isolate was susceptible to ampicillin, ceftriaxone, azithromycin, and trimethoprim-sulfamethoxazole while being resistant to ciprofloxacin and pefloxacin.

All the inpatients had fever defervescence with third-generation cephalosporin (ceftriaxone), although azithromycin was required after 7 days of initial ceftriaxone therapy in one infant. Nine patients who recovered healthy had a mean time to defervescence of 8.6 days and stayed in the hospital for a mean of 10 days.

## Discussion

Studies from developing countries demonstrating typhoid fever burden in children <5 years of age have been underappreciated, although the burden of disease in this subset of the population is high [12–14]. It is alarming to note that in the current study of children <12 years, 4.3% of enteric fever cases were in children <1 year of age, and 22% in those <2 years of age. A study from North India also suggested that about one-quarter of cases occur in children <5 years of age [15]. The present study highlights the importance vaccination in this target group and supports WHO recommendations to use TCVs for children <2 years of age.

Fever with abdominal symptoms was seen in most cases, with 46% of infants requiring inpatient admissions. And 54% cases being treated on outpatient basis. Significant morbidity and mortality rates are reported among children with typhoid fever [12, 16, 17]. In the present study, more than half (n = 14) of the patients had moderate anemia, and about 59% presented with abnormal liver enzyme levels. Furthermore, our data revealed a mortality rate of 7.6% (2 of 26) among infants presenting with enteric fever. A higher mortality rate of 18% was reported by Duggan and Beyer [18], with most deaths occurring in younger children. Suggested risk factors for typhoid fever are mainly within the household (contacts, sharing food from the same plate). In our study, only 39% of the infants were exclusively breastfed, while >50% were bottle fed, which suggests the need to investigate household risk factors in infants further. The mothers of the infants who were exclusive breastfed were questioned for clinical features of enteric fever in the recent past. None of the mothers reported any positive history indicating recent enteric fever. Two mothers reported a history of enteric fever 1 year before the birth of the infant. Fecal carriage of *S.* Typhi in the mothers could not be elicited.

The major contributor of enteric fever in our study was *S*. Typhi. However, another causative agent, *S*. Paratyphi, was also reported from multiple studies [19]. A noteworthy finding was that none of the enteric fever isolates from infants were multidrug resistant. The antimicrobial susceptibility pattern revealed that all isolates were susceptible to ceftriaxone, and none were susceptible to fluoroquinolones. Third-generation cephalosporin was used as first-line therapy for all the inpatients. The average fever defervescence time among the recovered patients was 8.6 days.

Our evidence of significant clinical illness among children <1 year of age with enteric fever stresses the need for an effective vaccine targeting the disease within this age group. The currently available vaccines, Ty21a live attenuated vaccine and parenteral Vi capsular polysaccharide vaccine, have low efficacy in such children. Infants presenting with fever and abdominal symptoms and living in high endemic areas, warrant a high suspicion of enteric fever in these cases. Less is known about the risk factors contributing to the spread of disease in infants. A detailed study focusing on risk factors for enteric fever among infants is needed to understand disease dynamics in this population.

To prevent disease and death related to enteric fever in children <1 year of age, it is imperative to devise public health strategies and preventive policies targeting enteric fever in this age group. Knowledge regarding disease burden in younger age groups will guide effective vaccination to target this subset of the population.

In conclusion, enteric fever caused by *S. enterica* species is a major public health problem, especially in resource-poor, endemic countries. The WHO’s Strategic Advisory Group of Experts on Immunization recommended prioritizing the programmatic use of TCVs in endemic and epidemic countries for children >6 months of age, for the control of typhoid. The quality, safety, and efficacy of TCVs have been recommended by WHO. In 2013, Typbar-TCV was first licensed in India for administration as a single dose in children aged ≥6 months and in adults up to age 45 years. A robust surveillance system for accurate estimation of disease burden among the infants and children is urgently needed for effective disease prevention and control. Data generated from in-depth studies will provide vital inputs for targeted immunization/vaccination of high-risk populations as a preventive intervention measure.

## Notes

***Acknowledgments.*** The work of all dedicated project staff and enrolled patients in the study is acknowledged.

***Author contributions***. K. S. and D. S. conceived and designed the study, developed the study protocol and analysis plan, and coordinated the project and data collection at the site. K. S. and D. G. analyzed the data and drafted the manuscript. All authors edited, reviewed, and approved the manuscript. K. S. had complete access to data and guarantees the manuscript.

***Supplement sponsorship.*** This supplement is sponsored by the Christian Medical College Vellore Association.

***Financial support.*** This study was part of the Surveillance System for Enteric Fever in India and was supported by the Bill & Melinda Gates Foundation (Investment ID grant INV-009497-OPP1159351).

***Potential conflict of interest.*** All authors: No reported conflicts. All authors have submitted the ICMJE Form for Disclosure of Potential Conflicts of Interest. Conflicts that the editors consider relevant to the content of the manuscript have been disclosed.
